# Purulent Streptococcus intermedius Pericarditis in the Setting of Histoplasma Mediastinal Lymphadenitis: A Case Report and Literature Review

**DOI:** 10.7759/cureus.62626

**Published:** 2024-06-18

**Authors:** Nathaniel Christian-Miller, Sayhaan Goraya, Patrick O'Hayer, Owen Albin, John Nicklas

**Affiliations:** 1 Internal Medicine, University of Michigan Hospitals, Ann Arbor, USA; 2 Cardiology, University of Michigan Hospitals, Ann Arbor, USA; 3 Infectious Disease, University of Michigan Hospitals, Ann Arbor, USA

**Keywords:** histoplasma in immunocompetent patient, constrictive pericarditis, tamponade physiology, pericardiectomy, purulent pericarditis

## Abstract

Purulent pericarditis is a rare and potentially life-threatening condition characterized by infection of the pericardial space. We describe a case of purulent bacterial pericarditis in a 41-year-old male with no significant medical or surgical history who had concomitant pulmonary *Histoplasma* infection. *Streptococcus intermedius *was the bacteria directly responsible for the pericardial infection, though co-infection with histoplasmosis likely predisposed him to develop purulent pericarditis. We hypothesize histoplasmosis caused mediastinal lymphadenopathy, facilitating contact between a necrotic lymph node and the pericardium and contiguous suppuration of bacteria to the pericardial space. We treated *S. intermedius* and *Histoplasma capsulatum* with ceftriaxone and amphotericin B, respectively.

Additionally, the patient presented in cardiac tamponade requiring emergent pericardiocentesis and drain placement. His course was also complicated by pericardial constriction. Cardiac magnetic resonance confirmed this, showing inflamed pericardium and abnormal septal motion with inspiration, and he had symptoms refractory to antimicrobials and anti-inflammatories. As such, he required pericardiectomy.

This case demonstrates maintaining suspicion for secondary infectious foci as a contributor to the pathogenesis of purulent pericarditis is important, as pulmonary histoplasmosis played a pivotal role in allowing *S. intermedius* to spread to the pericardium but was not the primary infection. It also highlights the multifaceted evaluation and management of purulent pericarditis, highlighting the role of echocardiography and emergent pericardial drainage if cardiac tamponade is present, the importance of targeted antimicrobial therapy, the superior ability of cardiac magnetic resonance to identify pericardial constriction as a sequela of purulent pericarditis, and indications for pericardiectomy.

## Introduction

Purulent pericarditis, defined as a local infection of the pericardial space with associated pus formation, is an uncommon but life-threatening diagnosis, with mortality of up to 20% to 30% even with treatment [1]. Commonly implicated organisms include *Staphylococcus aureus*, *Streptococcus pneumonia*, Viridans group Streptococci, *Haemophilus infuenzae*, anaerobic bacteria, and tuberculosis [2]. Pathways for pericardial infection include hematogenous spread, perforating chest injury, and contiguous extension from an intrathoracic process [3]. Predisposing factors have changed over time. In the pre-antibiotic era, a major risk factor was a primary underlying infection (e.g., pneumonia or endocarditis), with 86% of patients having a primary infection as opposed to 22% in the post-antibiotic era [3]. Risk factors in the modern era are more often comorbidities such as chronic kidney disease, immunosuppression, malignancy, or recent thoracic instrumentation [3].

Definitive diagnosis of purulent pericarditis itself rests on pericardial fluid analysis with the presence of grossly purulent pericardial fluid being 100% sensitive [2]. Less invasive diagnostic modalities that should be employed first include transthoracic echocardiogram (TTE) for quantifying pericardial fluid and evaluating for tamponade [4]. Cardiac tamponade is a potentially life-threatening compression of the cardiac chambers due to slow or rapid accumulation of fluid, pus, blood, or gas in the pericardium and is a rare but known sequelae of pericarditis [4]. Another potential complication is constrictive pericarditis which is caused by the development of granulation tissue in the pericardium leading to decreased elasticity and impaired ventricular filling. This is generally a chronic process and tends to occur late after untreated or recurrent episodes of acute pericarditis. Adjunctive imaging such as cardiac magnetic resonance (CMR) is useful for diagnosing constrictive pericarditis and can show classic features such as pericardial inflammation, suggested by late gadolinium enhancement of the pericardium, and real-time paradoxical septal motion with respiration [5].

We present a case of acute purulent pericarditis caused by *Streptococcus intermedius* in a patient with underlying pulmonary histoplasmosis, emphasizing pulmonary histoplasmosis as a unique risk factor in the antibiotic era. Concomitant infection with pulmonary histoplasmosis caused mediastinal lymphadenitis, thereby creating an environment where an enlarged, inflamed lymph node infected with *S. intermedius* was in proximity to the pericardium. We further emphasize the multimodal evaluation and treatment for purulent pericarditis, including expeditious drainage and targeted antimicrobials, the utility of CMR when there is a concern for progression to constrictive pericarditis, and the possible need for pericardiectomy if pericardial constriction develops. We also review prior cases of *S. intermedius* purulent pericarditis in the medical literature, showcasing how the organism has been associated with a higher incidence of tamponade and early constrictive pericarditis.

## Case presentation

**Figure 1:**
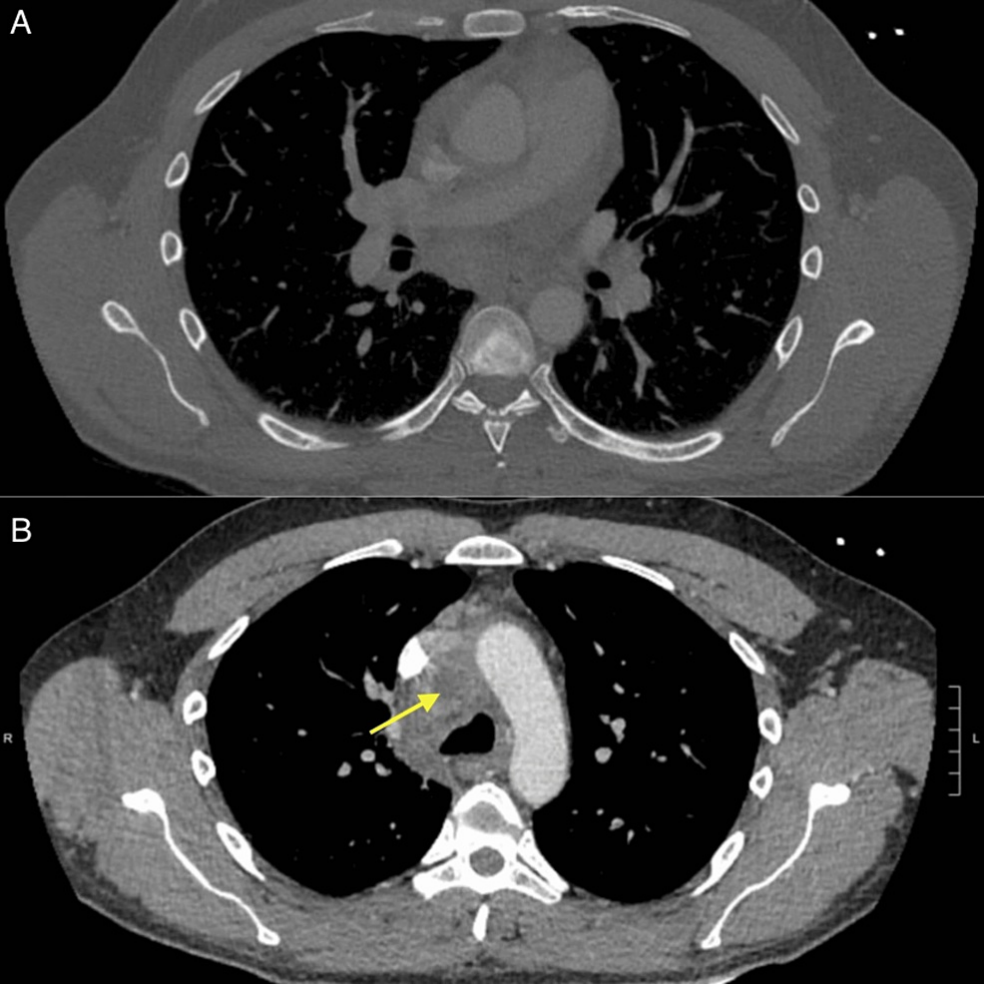
Chest CT showing (A) bilateral mediastinal and hilar adenopathy and (B) central area of low attenuation in the precarinal lymph node complex suggesting possible necrosis (yellow arrow). CT: computed tomography

A 41-year-old male with no significant past medical history presented to an outside hospital emergency department with several days of severe pleuritic chest pain, dyspnea, and night sweats. His vitals showed a blood pressure of 86/54 mmHg, a heart rate of 110 beats/minute, a respiratory rate of 21 breaths/minute, and a temperature of 36.5°C. Marked jugular venous distension was present. Initial laboratory work is shown in Table 1 and was notable for elevated inflammatory markers and neutrophilic leukocytosis. Thoracic computed tomography (CT) imaging showed trace pericardial effusion, multiple pulmonary nodules, and mediastinal and hilar adenopathy (Figure 1A) with a necrotic precarinal lymph node (Figure 1B).

**Table 1:** The patient’s presenting complete blood count, comprehensive metabolic panel, inflammatory markers, and cardiac biomarkers.

Test	Result	Reference range
Complete blood count and differential		
Hemoglobin	13.1 g/dL	13.5–17 g/dL
White blood cell count	18.2 K/µL	3.8–10.6 K/µL
Platelet count	375 K/µL	150–450 K/µL
Neutrophil, absolute	14.56 K/µL	1.80–7.70 K/µL
Lymphocytes, absolute	1.46 K/µL	1.10–4.00 K/µL
Monocytes, absolute	2.00 K/µL	0.00–0.80 K/µL
Eosinophils, absolute	0.18 K/µL	0.00–0.70 K/µL
Basophils, absolute	0.00 K/µL	0.00–0.20 K/µL
Comprehensive metabolic panel		
Sodium	130 mmol/L	135–145 mmol/L
Potassium	3.8 mmol/L	3.5–5.0 mmol/L
Chloride	95 mmol/L	98–111 mmol/L
Bicarbonate	26 mmol/L	21–35 mmol/L
Anion gap	9 mmol/L	3–13 mmol/L
Blood urea nitrogen	8 mg/dL	10–25 mg/dL
Creatinine	0.57 mg/dL	<1.13 mg/dL
Glucose	115 mg/dL	60–140 mg/dL
Bilirubin, total	0.8 mg/dL	<1.2 mg/dL
Bilirubin, direct	0.4 mg/dL	<0.0–0.3 mg/dL
Aspartate transaminase	24 IU/L	<35 IU/L
Alanine transaminase	26 IU/L	<40 IU/L
Alkaline phosphatase	90 IU/L	40–140 IU/L
Albumin	3.6 g/dL	3.7–4.8 g/dL
Calcium	9.5 mg/dL	8.2–10.2 mg/dL
Inflammatory markers		
C-reactive protein	27.1 mg/dL	<0.5 mg/dL
Erythrocyte sedimentation rate	29 mm/hour	<15 mm/hour
Cardiac biomarkers		
Troponin I	<4 ng/L	<15 ng/L
N-terminal-pro-hormone B-type natriuretic peptide	86 pg/mL	0–125 pg/mL

**Figure 2:**
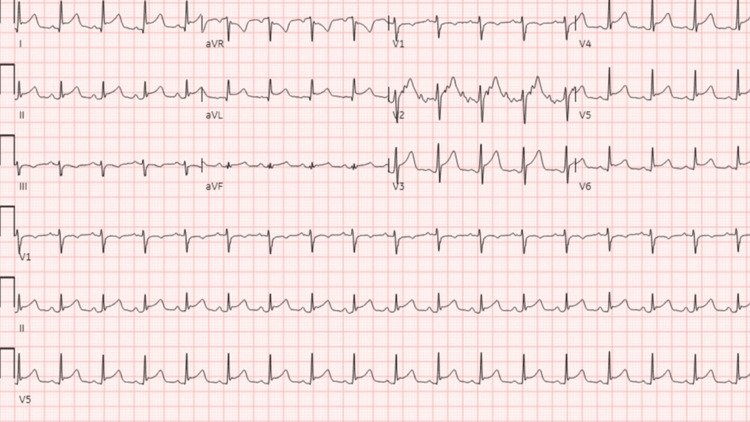
Admission EKG showing diffuse J-point/ST elevations in leads 1, avL, and V2-6. EKG: electrocardiogram

**Figure 3:**
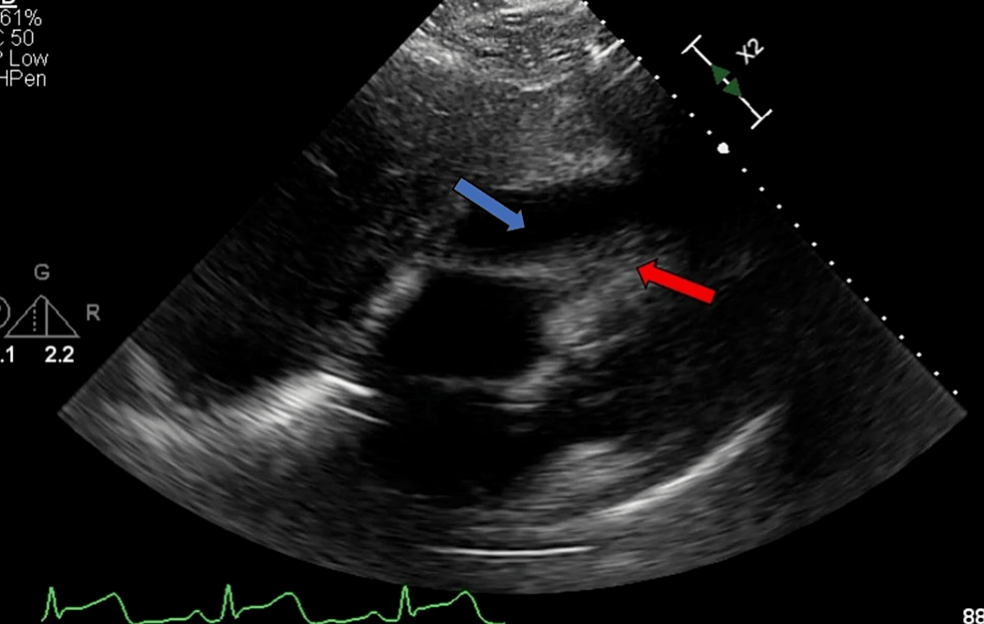
Initial TTE subcostal view showing pericardial effusion (blue arrow) and right ventricular collapse (red arrow) during diastole, consistent with tamponade physiology. TTE: transthoracic echocardiogram

Notably, his presenting electrocardiogram (EKG) showed diffuse J-point/ST elevations in leads 1, avL, and V2-6 and he was taken for percutaneous coronary intervention (Figure 2). However, angiography showed normal coronary arteries. TTE performed post-procedurally demonstrated a moderate pericardial effusion with right ventricular diastolic collapse, consistent with tamponade (Figure 3). He underwent emergent pericardiocentesis and pericardial drain placement with a return of 350 mL of purulent fluid and stabilization of his hemodynamics. Given his pleuritic chest pain, EKG findings, and new purulent effusion, he was clinically diagnosed with acute purulent pericarditis. Empiric antibiotics including vancomycin, cefepime, and doxycycline were started. The decision was made to transfer the patient to a tertiary care center, at which time pericardial gram stain and cultures were pending. Blood cultures and a respiratory viral panel at the outside hospital were negative.

On transfer, he was tachycardic but normotensive. A bedside echocardiogram showed resolution of tamponade and decreased effusion. His early course following transfer was notable for new, intermittent high-grade fevers and persistent chest pain. Colchicine was started at 0.6 mg twice daily. Repeat laboratory work demonstrated an increasing leukocytosis and elevated high-sensitivity troponin (Table 2). Troponin levels peaked at 43 ng/L and normalized over the next two days.

**Table 2:** The patient’s laboratory work upon transfer.

Test	Result	Reference range
Complete blood count and differential		
Hemoglobin	10.8 g/dL	13.5–17.0 g/dL
White blood cell count	28.8 K/µL	4.0–10.0 K/µL
Platelet count	363 K/µL	150–400 K/µL
Neutrophil, absolute	24.9 K/µL	1.5–7.2 K/µL
Lymphocytes, absolute	1.6 K/µL	1.2–4.0 K/µL
Monocytes, absolute	2.20 K/µL	0.1–1.1 K/µL
Eosinophils, absolute	0.00 K/µL	0.00–0.50 K/µL
Basophils, absolute	0.10 K/µL	0.00–0.20 K/µL
Comprehensive metabolic panel		
Sodium	139 mmol/L	136–146 mmol/L
Potassium	4.4 mmol/L	3.5–5.1 mmol/L
Chloride	106 mmol/L	98–108 mmol/L
Bicarbonate	24 mmol/L	20–31 mmol/L
Anion gap	9 mmol/L	2–13 mmol/L
Blood urea nitrogen	8 mg/dL	8–20 mg/dL
Creatinine	0.69 mg/dL	0.70–1.30 mg/dL
Glucose	115 mg/dL	70–180 mg/dL
Bilirubin, total	0.7 mg/dL	0.2–1.2 mg/dL
Aspartate transaminase	15 IU/L	<34 IU/L
Alanine transaminase	23 IU/L	<10–49 IU/L
Alkaline phosphatase	75 IU/L	40–116 IU/L
Albumin	3.3 g/dL	3.5–4.9 g/dL
Calcium	7.8 mg/dL	8.6–10.3 mg/dL
Cardiac biomarkers		
High-sensitivity troponin T	25 pg/mL	0–19 pg/mL
B-type natriuretic peptide	65 pg/mL	≤100 pg/mL

The patient denied intravenous drug use, immunocompromising conditions or medications, or a history of thoracic instrumentation or radiation. He did describe recent travel to Kentucky with multiple zoonotic exposures, including temporary residence at a venue with free-range chickens and bats, as well as a visit to a petting zoo. He also reported work as an amateur arborist.

An extensive infectious workup was obtained to evaluate for the cause of pericarditis. Blood and fungal cultures were normal. Human immunodeficiency virus (HIV), tuberculosis, and syphilis were considered, although HIV, quantiferon gold testing, acid-fast bacilli pericardial fluid cultures, and rapid plasma regain testing, respectively, were negative. There was high suspicion for histoplasmosis given his exposure history, mediastinal/hilar lymphadenopathy, and lack of therapeutic response to broad-spectrum antibiotics and liposomal amphotericin B was started. At this time, a *Histoplasma* serum antigen was pending.

**Figure 4:**
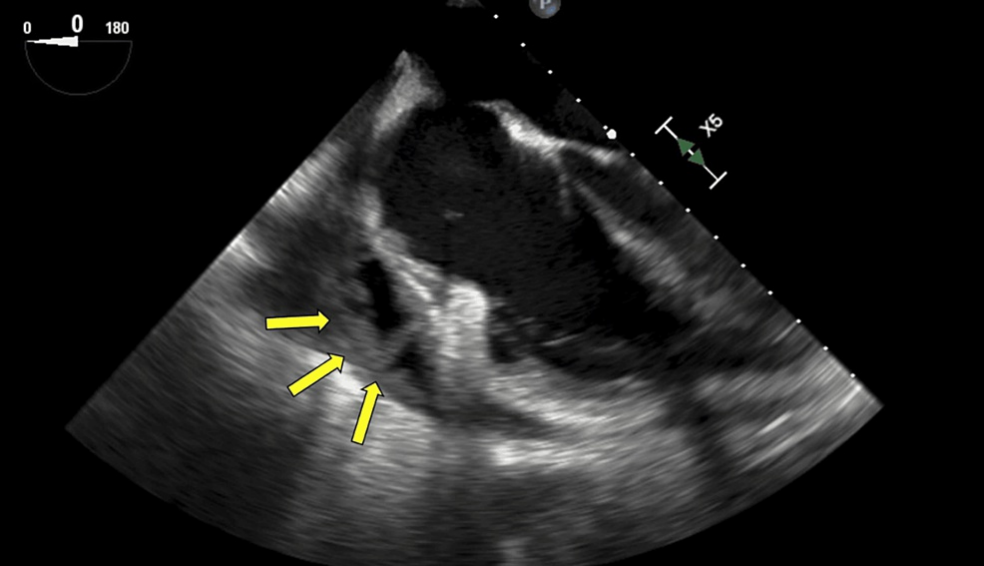
Focused right atrial/right ventricular four-chamber view on TEE showing pericardial effusion containing loculations and fibrinous strands (yellow arrows). TEE: transesophageal echocardiogram

**Figure 5:**
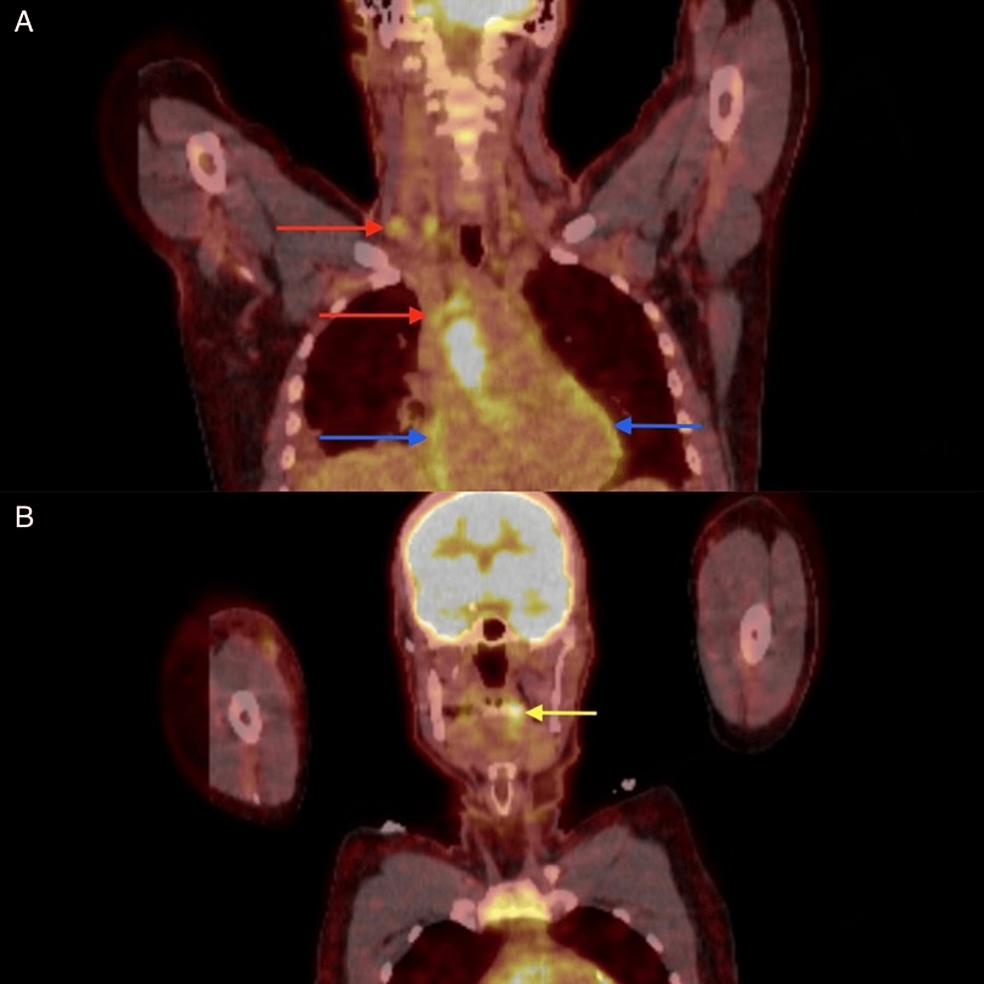
PET scan showing (A) hypermetabolic activity in the cervical/mediastinal lymph nodes (red arrows), pericardial fluid (blue arrows), and (B) left palatine tonsil (yellow arrow). PET: positron emission tomography

Notably, pericardial fluid cultures at the outside hospital returned positive for *S. intermedius* two days after transfer, and antibiotics were narrowed to ceftriaxone. The patient’s pericardial fluid analysis is shown in Table 3. Source evaluation included a transesophageal echocardiogram (TEE) to assess for endocarditis which showed no intracardiac vegetation but did show evidence of fibrinous material and loculated fluid in the pericardial space (Figure 4). A dental examination was normal. A nuclear medicine positron emission tomography (NM PET) scan demonstrated hypermetabolic mediastinal and cervical lymph nodes, pericardial fluid (Figure 5A), and asymmetric 18-fluoro-deoxyglucose (FDG) uptake in the left palatine tonsil (Figure 5B). Pulmonary medicine was eventually consulted for bronchoscopy, as it was thought the previously seen necrotic precarinal node may have created a fistulizing tract for bacterial seeding of the pericardium; however, his airway examination was normal.

**Table 3:** The patient’s presenting pericardial fluid analysis.

Test	Result	Reference range
Lactate dehydrogenase	1,643 U/L	<200 U/L
Glucose	<10 mg/dL	60–80 mg/dL
Adenosine deaminase	10 U/L	<40 U/L
Protein	5.5 g/dL	<3.0 g/dL
pH	7.5	6.8–7.5
Red blood cells	11,000 cells/mm^3^	None seen
White blood cells	144,440 cells/mm^3^	<500 cells/mm^3^
Neutrophils	92%	<25%
Lymphocytes	6%	Not established
Monocytes	2%	Not established
Clarity	Turbid	Clear
Color	White	Clear to straw yellow
Bacterial culture	*Streptococcus intermedius*	No organisms isolated
Acid-fast bacilli culture	No organisms isolated	No organisms isolated
Fungal culture	No organisms isolated	No organisms isolated

**Figure 6:**
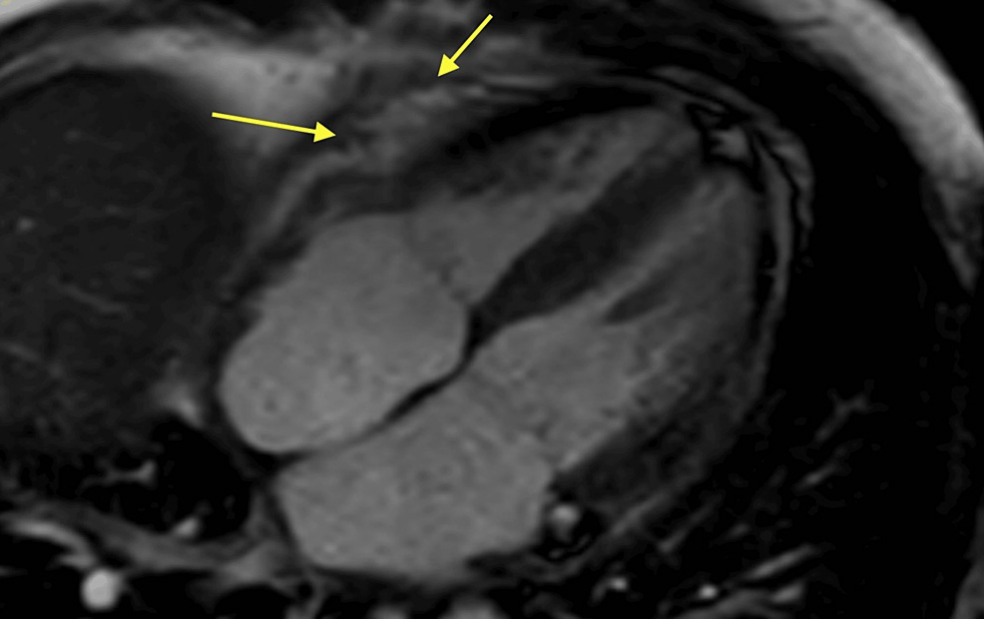
CMR showing enhancing, thickened pericardium consistent with inflammatory constrictive pericarditis. CMR: cardiac magnetic resonance

Five days after transfer, his serum antigen test for *Histoplasma* returned positive and he was given a loading dose of itraconazole. Excluding his pericardial fluid analysis, the patient’s infectious evaluation is shown in Table 4. In the following days, his fevers resolved, his pericardial drain output was less than 10 mL per day, and his pain and dyspnea improved. CMR showed normal systolic function and myocardium but also paradoxical diastolic septal motion during inspiration and enhancing, thickened pericardium consistent with constrictive pericarditis (Figure 6). Despite this, given his clinical stability and apparent improvement in his symptoms, it was felt reasonable to trial medical management with follow-up CMR in eight weeks and plans for pericardiectomy if this showed persistent constriction. His drain was removed after a successful clamping trial. His antimicrobial regimen at discharge included linezolid 600 mg twice daily for four weeks and a prolonged course of itraconazole 200 mg daily for three months. He was also discharged on colchicine 0.6 mg twice daily for three months and ibuprofen 800 mg three times daily for two weeks.

**Table 4:** The patient’s infectious evaluation excluding pericardial fluid cultures.

Test	Result	Reference range
Histoplasma serum antigen	Positive	Negative
Quantiferon TB	Negative	Negative
Acid-fast bacilli culture	No acid-fast bacilli isolated	No acid-fast bacilli isolated
Aerobic and anaerobic blood cultures	No growth	No growth
Fungal blood culture	No growth	No growth
Human immunodeficiency virus antigen-antibody	Non-reactive	Non-reactive
Rapid plasma reagin	Non-reactive	Non-reactive
Respiratory viral panel	None detected	None detected

**Figure 7:**
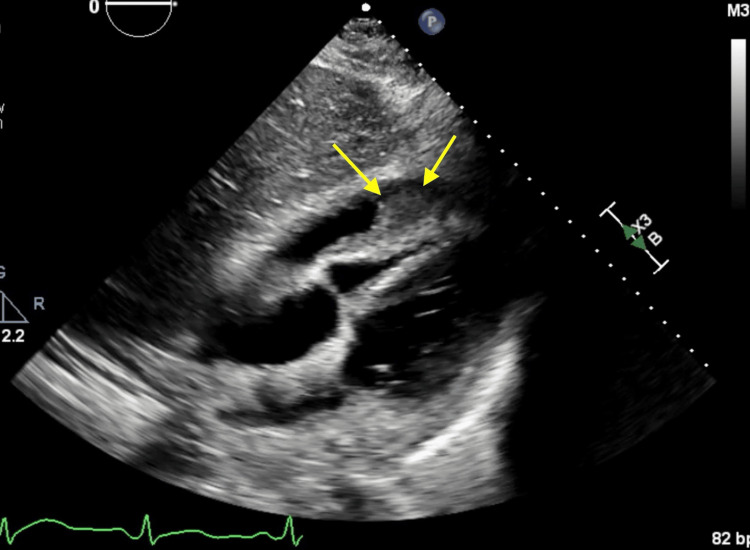
Readmission TTE showing persistent loculations and fibrinous material in the pericardial effusion (yellow arrows). TTE: transthoracic echocardiogram

However, one day after discharge he presented again with recurrent fevers and chest pain. A TTE showed increased loculated pericardial fluid and fibrinous material and evidence of ongoing constrictive pericarditis (Figure 7). Given his persistent constrictive physiology and pericardial inflammation despite medical management, he ultimately underwent pericardiectomy with intra-procedural findings of thick, inflamed pericardium and fibrinous debris in the pericardial space. On discharge, his previous antimicrobial regimen of linezolid and itraconazole was continued and close follow-up with cardiology, cardiac surgery, and infectious disease was arranged.

## Discussion

We describe a case of purulent streptococcal pericarditis which was likely abetted by underlying pulmonary infection with *Histoplasma*. Mechanistically, we believe the patient had either oral pharyngitis or tonsillitis from *S. intermedius* permitting lymphatic spread. In the setting of mediastinal lymphadenitis caused by pulmonary histoplasmosis, this created a route for *S. intermedius* to contiguously suppurate to the pericardium from a proximally infected lymph node.

Purulent pericarditis is rarely seen in the antibiotic era, with one retrospective study reporting 33 cases among a population of 593,600 [2]. Presenting characteristics include fever, chills, tachycardia, and, more rarely, chest pain and the presence of a friction rub [2,4]. The presence of pericardial effusion with macro- or microscopic pus is diagnostic, and cardiac tamponade is observed in 42% to 77% of patients based on previous case series and can cause life-threatening hemodynamic instability [1,2]. Laboratory findings are nonspecific and include leukocytosis, anemia, and elevated inflammatory markers [6].

Regarding pathogenesis, one mechanism by which purulent pericarditis can develop is spread from intrathoracic processes [3], and pulmonary histoplasmosis commonly manifests with hilar and mediastinal adenopathy [7], as seen on this patient’s chest imaging. Further, while *S. intermedius* is part of commensal oral flora and is generally considered low virulence, it has been reported as a cause of purulent pericarditis in immunocompetent hosts before [8,9]. Given the asymmetric hypermetabolic activity in the left palatine tonsil on PET, the most plausible explanation is oropharyngeal infection by *S. intermedius* caused transient bacteremia, permitting infection of a lymph node close to the pericardial space. Pulmonary histoplasmosis contributed to this by causing mediastinal lymphadenitis, bringing one or several lymph nodes near and creating a route for *S. intermedius* to seed the pericardium. Another possibility is direct hematogenous seeding of the pericardium by *S. intermedius*, though this is less likely as the patient’s blood cultures were repeatedly negative even before antibiotic initiation at the outside hospital.

*S. intermedius* is a member of the *Streptococcus milleri* group, which also includes *Streptococcus constellatus* and *Streptococcus anginosus*. They are traditionally classified as oral commensal bacteria but have been associated with opportunistic infections including abscesses affecting the cardiovascular, pulmonary, gastrointestinal, and central nervous systems [10]. *S. intermedius* has been specifically implicated in brain, liver, lung, and orofacial abscesses, and less frequently pericarditis [10]. These organisms are also capable of severe disseminated infection. The pathogenesis of multiple cases of *S. intermedius* bacteremia has been linked to disruption of the oral mucosal barrier in the context of either oral infection or instrumentation, allowing an invasion of the underlying tissue [10].

Overall, reports of purulent pericarditis due to *S. intermedius* are rare. We identified six previously reported cases on a search of the literature, with notable features summarized in Table 5 [9,11-15]. To our knowledge, this represents the seventh case of bacterial pericarditis caused by *S. intermedius*. Prototypical risk factors were absent in 3/7 (43%) cases [9,13,15]. Cardiac tamponade was present in a majority of cases, occurring in 5/7 (71%) patients [9,11-13,15]. Pericardiocentesis was the initial method of pericardial drainage in all but one case. Notably, three patients developed pericardial constriction [9,13,15] and two underwent pericardiectomy [9,15] during their initial presentation. Only one case identified an obvious mechanism (a fistulizing tract due to esophageal carcinoma) for *S. intermedius* to spread to the pericardial space [12] while the remainder found no conspicuous source. Collectively, these reports show a majority of patients with reported occurrences of *S. intermedius* purulent pericarditis were immunocompetent and developed tamponade. They also demonstrate a nontrivial incidence of early-onset constrictive pericarditis requiring more invasive pericardial drainage such as pericardiectomy, similar to our case. While this is a limited sample, these reports suggest *S. intermedius* purulent pericarditis can occur in the absence of classic risk factors and may be associated with greater morbidity, including a higher incidence of tamponade and earlier progression to constrictive pericarditis.

**Table 5:** Summary of characteristics of previously reported cases of S. intermedius purulent pericarditis.

Reference	Past medical history	Tamponade present	Initial method of pericardial drainage	Evidence of constrictive pericarditis	Adjunctive surgical management	Alive at discharge
Khan et al., 2018 [9]	None	Yes	Pericardiocentesis	Yes	Pericardiectomy	Yes
Denby et al., 2017 [11]	None	Yes	Pericardiocentesis	No	Pericardial window	Yes
Muto et al., 1999 (1) [12]	Esophageal carcinoma	Yes	Pericardiocentesis	No	None	No
Muto et al., 1999 (2) [12]	Esophageal carcinoma	No	Pericardiocentesis	No	None	No
Presnell et al., 2014 [13]	None	Yes	Pericardial window	Yes	None	Yes
Rougé et al., 2016 [14]	Type 2 diabetes mellitus	No	Pericardiocentesis	No	None	Yes
Tigan et al., 2015 [15]	Bronchiectasis	Yes	Pericardiocentesis	Yes	Pericardiectomy	Yes

Definitive diagnosis of purulent pericarditis requires pericardial fluid analysis and samples should be sent for gram, acid-fast bacilli, and fungal stains; bacterial and fungal cultures; and a cell count with differential [4]. This can assess for other known causes of purulent pericarditis including aerobic/anaerobic bacteria, tuberculosis, and *Candida*. Fluid studies typically show neutrophilic predominance, elevated lactose dehydrogenase, low glucose, and high protein [4]. While we hypothesize histoplasmosis enabled pericardial seeding by *S. intermedius*, we do not suspect it was the primary cause of this patient’s pericarditis. *Histoplasma* pericarditis is caused by a hypersensitivity reaction to yeast within the mediastinal lymph nodes and is associated with hemorrhagic, lymphocyte-predominant pericardial fluid [7,16,17] which was inconsistent with the fluid analysis here.

As in our case, purulent pericarditis is often accompanied by EKG findings consistent with acute pericarditis (i.e., diffuse ST elevations and PR depression throughout the precordial and limb leads) but may be normal in up to a third of presentations [4]. Echocardiography can quickly and noninvasively quantify pericardial fluid and assess for tamponade [4]. CMR has emerged as a highly useful aid for evaluating pericardial disease through its ability to characterize pericardial inflammation, effusion, myocardial involvement, and constrictive physiology [5]. Of the various cardiac imaging modalities, it is helpful to assess for progression to constrictive pericarditis through its superior ability to assess the degree of pericardial thickening, the presence of inflammation with contrast enhancement, and to identify paradoxical diastolic septal motion with inspiration, which is a highly specific feature for constriction [5]. It may also have an especially important role in differentiating pericardial effusions based on T1 and T2-weighted signal intensities; purulent effusions will typically manifest with low T1 but high T2 signal intensity [5].

Management consists of pericardial drainage and antimicrobials [6]. Pericardiocentesis is often the most expeditious method of draining the pericardial space but more invasive and complete options include pericardiotomy, pericardiectomy, and video-assisted thoracic surgery [6]. Notably, constrictive pericarditis can arise if pericardiocentesis is employed as the primary drainage method [2], which likely occurred in this case. Pericardiectomy entails removing as much of the constricting visceral pericardial layers as feasible while not damaging the phrenic nerves [4]. It is the standard of care for pericardial evacuation in cases of chronic constrictive pericarditis refractory to medical management but also considered in the setting of loculated or reaccumulating pericardial effusion [4,18]. We suspect the reason this patient presented again so quickly after his initial discharge was because he had persistent purulent pericarditis inadequately treated with pericardiocentesis and drain placement alone. This has been documented in prior case reports, especially when loculations and fibrinous material are present in pericardial fluid [19]. As seen on this patient’s TEE, both fibrin and loculations were observed in the pericardial space before pericardial drain removal and had an increased burden on readmission echocardiography.

Antibiotics should be directed against the causative organism. In this case, it was important to treat both the bacteria cultured from the patient’s pericardial fluid and the histoplasmosis we felt predisposed him to infection. Treatment of *Histoplasma* usually consists of itraconazole but amphotericin B is recommended in severe or disseminated cases [20]. As with other causes of pericarditis, nonsteroidal anti-inflammatory drugs and colchicine may prevent recurrence [4].

## Conclusions

Purulent pericarditis carries high mortality and requires a multifaceted approach to evaluation and management. Urgent recognition with pericardial fluid analysis and treatment with drainage and antibiotics is imperative. Here, it was important to treat both *S. intermedius* and the patient’s pulmonary histoplasmosis as we suspect the latter was a secondary infection that enabled the primary organism, *S. intermedius*, to suppurate to the pericardium. Additionally, echocardiography is indicated for detection and quantification of pericardial fluid and CMR is highly useful for evaluating the degree of pericardial disease and progression to constrictive pericarditis. When constrictive physiology develops, symptoms are refractory to medical management, and/or when the pericardial effusion reaccumulates or becomes loculated, more complete methods of pericardial drainage such as pericardiectomy may be necessary. In addition to highlighting the comprehensive treatment approach necessary to care for patients with purulent pericarditis, our case highlights the uniqueness of concurrent *Histoplasma* infection as a key risk factor in the antibiotic era and adds to the limited literature suggesting *S. intermedius* may be associated with more severe presentations of purulent pericarditis.
